# The efficacy of addition of dexmedetomidine to intrathecal bupivacaine in lower abdominal surgery under spinal anesthesia

**DOI:** 10.22088/cjim.10.2.142

**Published:** 2019

**Authors:** Milad Minagar, Ebrahim Alijanpour, Ali Jabbari, Seyed Mozaffar Rabiee, Nadia Banihashem, Parviz Amri, Mehrafza Mir, Mohammad Taghi Hedayati Goodarzi, Mohammad Esmaili

**Affiliations:** 1 Department of Anesthesiology and Critical Care Medicine, Tehran University of Medical Sciences, Tehran, Iran; 2 Department of Anesthesiology and Critical Care Medicine, Babol University of Medical Sciences, Babol, Iran; 3 Department of Anesthesiology and Critical Care Medicine, Golestan University of Medical Sciences, Gorgan, Iran; 4 Department of Cardiology, Babol University of Medical Sciences, Babol, Iran

**Keywords:** Dexmedetomidine, Bupivacaine, Lower abdominal surgery, Spinal anesthesia

## Abstract

**Background:**

Spinal anesthesia is the common choice for anesthesia in lower abdomen surgery and intrathecal adjutants have gained popularity with the aim of prolonging the duration of block, quality of block and post operation pain control. The purpose of this study was to evaluate the effects of adding dexmedetomidine to hyperbaric bupivacaine in lower abdominal surgery under spinal anesthesia. The main outcomes were considered pain score, duration of analgesia, hemodynamic changes and adverse side effects like nausea and vomiting.

**Methods:**

This double-blind randomized clinical trial was conducted on one hundred patients between 18 to 65 years old scheduled for lower abdominal surgery. Fifty patients were randomly allocated to receive either 12.5mg hyperbaric bupivacaine (2.5cc) plus 5µgr dexmedetomidine (0.5cc) intrathecally while fifty patients received either 12.5mg hyperbaric bupivacaine (2.5cc) and 0.5cc Saline 0.9% intrathecally.

**Results:**

Vital sign parameters like heart rate, blood pressure and oxygen saturation levels were registered in the normal range in both groups. The average duration of the onset of pain (230±86 min) in bupivacaine group was significantly (p≤0.000) less than dexmedetomidine group (495±138 minutes). The severity of pain at all times in dexmedetomidine group was significantly (p<0.05) less than bupivacaine group. The severity of shivering and the number of patients who needed treatment for nausea and vomiting in dexmedetomedine group has been less in comparison to bupivacaine.

**Conclusion:**

We concluded that intrathecal dexmedetomidine increases the duration of analgesia and reduces postoperative pain without changes in the hemodynamic parameters and adverse side effects. It can be considered as an appropriate adjuvant to intrathecal local anesthetics for lower limb surgeries.

Postoperative pain is a major concern for patients and postoperative pain management is considered as a part of perioperative care (1). Lower abdominal surgeries became popular under neuraxial block because it is a cost-effective method and an easy-to-perform technique (2, 3). Spinal anesthesia is still the first option, due to having rapid onset, superior blockade with a lower failure rate but it is associated with such drawbacks as shorter duration of the block, short-term anesthetic effect, and the onset of pain after the absorption of the drug (2). Different adjuvant can be used, along with local anesthetics for spinal anesthesia to relieve pain during operation and provide long-term postoperative analgesia (3). 

Alpha receptor agonists are added to local anesthetics as an adjuvant to improve their effects and reduce the required dose of anesthetics (4). Dexmedetomidine is a selective α2 agonist that is 1,600 times more selective for the alpha-2 receptor than the alpha-1 receptor (1, 5). This drug has anti-anxiety, sedative, analgesic, and sympatholytic effects with minimum respiratory depression (6-8). This study aimed to investigate the effect of intrathecal dexmedetomidine with local anesthetic on analgesic duration during and after the operation.

## Methods

In this double-blind randomized controlled trial study in 2013, 100 patients aged between 18 and 65 years, in ASA (American Society of Anesthesiologists) class I, II and candidate for elective lower abdominal surgery, such as prostatectomy, varicocelectomy, hysterectomy, and inguinal herniorrhaphy, were included after obtaining their informed consent in Shahid Beheshti Hospital in Babol. This study was registered in the Clinical Trial Registration Center of Iran under the code IRCT201310293305N6. Patients with a history of cardiovascular diseases, dyspnea, diabetes, and renal or liver complications, patients with a history of taking narcotics, antiepileptic drugs, antipsychotics, and/or analgesics, and patients with a history of consuming alcohol were excluded. 

In addition, patients with severe pain at the operation site during surgery, which necessitated general anesthesia, were removed from the study. Sample size calculation was performed on Hong study’s result (9) and its related formula for sample size in a=0.05 and β=0.8. Patients were divided into two 50-member groups using simple random sampling technique by a resident anesthesiologist. For allocation concealment, we used opaque pocket. The case group received dexmedetomidine+bupivacaine (Marcaine); whereas, the control group only received Marcaine. After transferring the patients to the procedure table, standard monitoring systems, including ECG, NIBP, and SPO2 were connected to them. 

Then, Ringer's serum (5 ml per kilogram body weight) was administered through intravenous cannulation. After measuring HR, BP, and SPO2 (as basic measurements), patients were placed in a sitting position, and prepping and draping were conducted. Then, the case group received intrathecal injection of 2.5 ml of bupivacaine (0.5%) and 0.5 ml of dexmedetomidine (5μg); whereas, the control group received intrathecal injection of 2.5ml of bupivacaine (0.5%) and 0.5 ml of normal saline at lumbar segments 3-4 (L3-L4) or 4-5 (L4-L5), using 25-gauge needles (B-Braun Co.). After placing the patients in a supine position, all of them received a venturi mask with an oxygen flow between 5-6 ml per minute. In addition, 1/1-2 mg of intravenous midazolam, as a sedative, was administered. Both the patient and outcome assessor were blind to the type of administering intrathecal drug. 

Hemodynamic parameters of the patient in the 0 (baseline), 5th, 10th, 15th, 30th, 60th, and 120th minutes and 6th, 12th, 18th, and 24th hours were measured and recorded. Patients with systolic blood pressure < 90 received 5-10 mg of ephedrine. Moreover, patients with HR < 60 per minute received 0.5 mg of atropine. 

During surgery and recovery, the patients were examined and were categorized in terms of shivering, which was graded as follows: 0 = no shivering, 1= piloerection or peripheral vasoconstriction, but no visible shivering, 2= shivering in only one muscle group, 3= shivering in more than one muscle group but not generalized, and 4 = generalized shivering involving the whole body. Patients with extreme and generalized shivering (grade 4) were treated with pethidine 25 mg. In addition, patients were graded between 0-10 over 24 hours after the surgery in terms of postoperative onset and severity of pain was based on a visual analog scale (VAS). 

The VAS pain grading system is as follows: 0 = without pain, 1-2 = slight pain, 3-4 = mild pain, 5-6 = moderate pain, 7-8 = severe pain and 9-10 = the worst pain. If pain grade of a patient was greater than 4 and/or the patient ask for analgesics because of having pain, pethidine 0.5 mg per kilogram body weight (maximum dose=30 mg) was administered. Additionally, patients were investigated in terms of nausea and vomiting over 24 hours after the surgery. 

Intravenous metoclopramide 10 mg was administered to patients with such problems. In this study, severity of pain at different times and average duration of the onset of pain as primary outcomes and shivering, nausea, and vomiting post operation as secondary outcomes were recorded. Patients lost to follow-up were excluded from the study completely. Data were analyzed using chi-square test, Fisher’s exact test, t-test, Mann-Whitney U test, and repeated measures in SPSS 16. Furthermore, p<0.05 was considered to be significant. 

## Results

**Figure 1:**
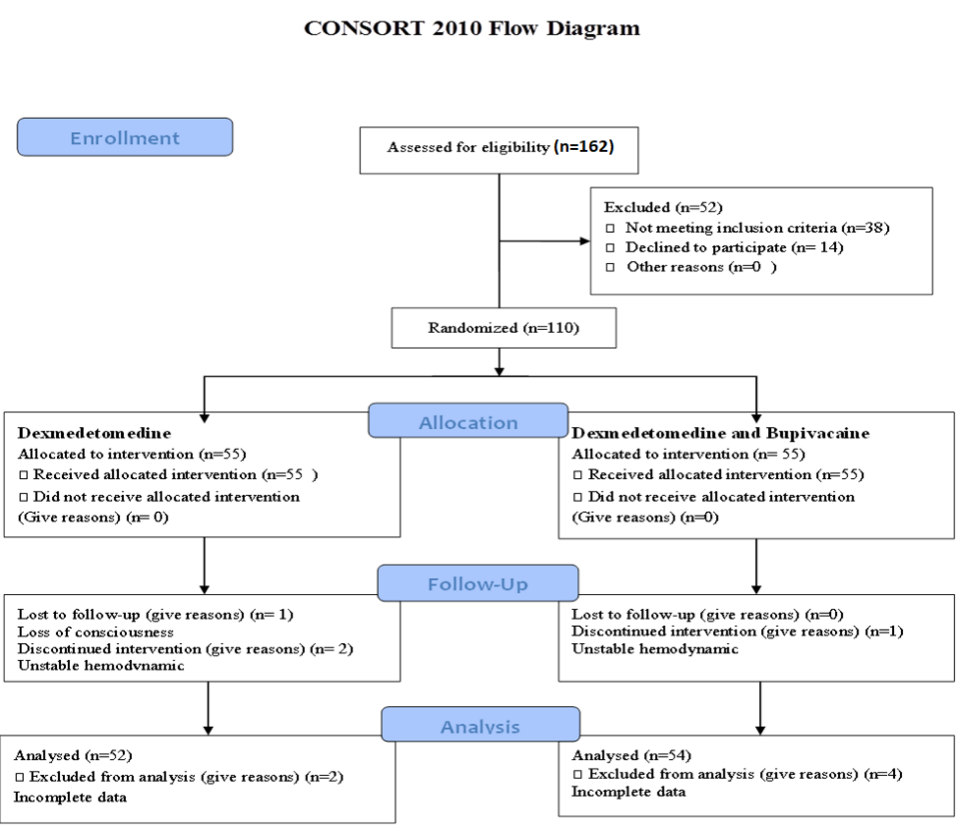
Consort flowchart

This study was conducted on 100 patients, who were equally divided into dexmedetomidine plus bupivacaine and bupivacaine groups. There were 40 (80%) women and 10 (20%) men in the bupivacaine group and 22 (44%) women and 28 (56%) men in the dexmedetomidine group (figure 1). 

The mean and standard deviation of other demographic characteristics of the patients in both groups are presented in table 1. According to tables 2-5, hemodynamic parameters may be statistically but not clinically significant in the beginning or some other times, and changes are less than 20% of the baseline. The mean and standard deviation of oxygen saturation of the patients at different times are presented in table 5.

The mean and median of severity of pain at different times for both groups is presented in table 6. Average duration of the onset of pain in the bupivacaine group was 230±86 minutes, which was significantly lower than the dexmedetomidine+bupivacaine group with the mean of 495±138 (p=0.000). Moreover, 40 (80%) patients in the bupivacaine group and 39 (78%) patients in the dexmedetomidine+bupivacaine group received pethidine (p>0.05).

 The mean pethidine intake in the bupivacaine group and dexmedetomidine+bupivacaine group was 87.5 mg and 33.6 mg, respectively (P=0.000). 

The shivering, nausea, and vomiting conditions of the patients in both groups are presented in table 6. The mean severity of shivering in the bupivacaine group and dexmedetomidine+bupivacaine group was 1.4 and 0.9, respectively (P=0.377). Moreover, eight patients in the bupivacaine group received pethidine for shivering treatment. Eighteen patients in the bupivacaine group and 12 patients in the dexmedetomidine+bupivacaine group received metoclopramide 10 mg as an antiemetic (P=0.257). Nineteen patients in the dexmedetomidine+bupivacaine group had nausea and vomiting, out of which seven patients had mild nausea and did not need treatment; whereas, all 18 patients in the bupivacaine group received metoclopramide for having severe nausea and vomiting.

**Table 1:** Mean and standard deviation of demographic information of patients in both groups

**Group** **variable**	**Bupivacaine (50 patients)**	Dexmedetomidine+bupivacaine (50 patients)	**P-value** *
Age (year)	44.4±14.5	44.3±13	0.977
Height (cm)	169±7.8	171.9±6.8	0.048
Weight (kg)	72.1±12.1	73.3±5.4	0.547
BMI (Kg/m3)	25.2±4.3	24.9±2.1	0.626

BMI: Body mass index

* Independent sample t-test

**Table 2:** Mean and standard deviation of heart rate in both groups

**Group** **time**	**Bupivacaine** **(50 patients)**	**Dexmedetomidine** **+bupivacaine** **(50 patients)**	**Mean difference**	**CI 95%**		**P-value** *
				**Lower**	**Upper**	
Zero	79.4±18.5	88.6±10.4	-9.2	-15.1	-3.2	0.003
5th minute	76.4±18.1	85.4±11.6	-9.0	-15.0	-2.9	0.004
10th minute	74.4±17.1	75.8±8.2	-1.4	-6.7	3.9	0.605
15th minute	72.7±16.9	69.7±8.8	3.0	-2.3	8.3	0.27
30th minute	71.4±16.1	69.7±8	4.3	-0.7	9.3	0.096
60th minute	69.4±12.5	67.8±6.5	1.6	-2.3	5.6	0.409
120th minute	70.7±12	68.2±5.9	2.5	-1.2	6.2	0.193
6th hour	74.6±7.8	70.8±5.2	3.7	1.0	6.3	0.006
12th hour	74.3±5.7	73.2±5.4	1.1	-1.1	3.3	0.328
18th hour	72.2±6.6	74.1±4.4	-1.9	-4.1	0.3	0.097
24th hour	76.4±5.2	74.7±5.9	1.7	-0.5	3.9	0.128
P-value	0.001	0.000				

* Independent sample t-test

**Table 3:** Mean and standard deviation of systolic blood pressure in both groups

**Group** **time**	**Bupivacaine** **(50 patients)**	**Dexmedetomidine** **+bupivacaine** **(50 patients)**	**Mean difference**	**CI 95%**		**P-value** *
				**Lower**	**Upper**	
Zero	139.1±13.7	147.1±14.1	-8.0	-13.5	-2.4	0.005
5th minute	127.9±15	137.8±12.5	-9.9	-15.4	-4.4	0.001
10th minute	122.6±14.7	129.4±10.1	-6.8	-11.8	-1.7	0.009
15th minute	115.7±16.1	122.7±10.7	-7.0	-12.4	-1.5	0.012
30th minute	115±16	117.5±11.6	-2.4	-8.0	3.0	0.379
60th minute	115.4±15.1	118.2±7.8	-2.8	-7.6	1.9	0.236
120th minute	115.7±12.2	118.9±7.6	-3.1	-7.2	0.8	0.124
6th hour	122±10.8	124.3±8.5	-2.2	-6.1	1.5	0.246
12th hour	124.2±13.3	128±8.1	-3.7	-8.1	0.6	0.094
18th hour	123.3±13.9	129±9.3	-5.6	-10.3	-0.9	0.019
24th hour	126.1±13.6	129.3±7.8	-3.1	-7.5	1.2	0.159
P-value	0.000	0.000				

* Independent sample t-test

**Table 4:** Mean and standard deviation of diastolic blood pressure in both groups

**Group** **time**	**Bupivacaine** **(50 patients)**	**Dexmedetomidine+bupivacaine** **(50 patients)**	**Mean Difference**	**CI 95%**		**P-value** *
				**Lower**	**Upper**	
Zero	88.2±8.9	81.4±9	6.7	3.1	10.2	0.000
5th minute	79.4±11.9	76.2±8.6	3.2	-0.9	7.3	0.126
10th minute	74.8±11.7	72.2±7.9	2.6	-1.3	6.6	0.192
15th minute	71.9±11.9	68.6±8.1	3.3	-0.7	7.3	0.105
30th minute	71.4±13.5	65.6±7.8	5.9	1.4	10.3	0.009
60th minute	71±11.9	64.6±6.1	6.3	2.5	10.1	0.001
120th minute	71.3±8.7	64.3±6.1	6.9	3.9	9.9	0.000
6th hour	73.4±9.1	67.2±5.6	6.1	3.1	9.1	0.000
12th hour	75±8.3	69±6	6.0	3.1	8.9	0.000
18th hour	74.3±9.1	69±7	5.3	2.0	8.5	0.002
24th hour	76.8±9.2	70.8±6.9	6.1	2.9	9.4	0.000
P-value	0.000	0.000				

* Independent sample t-test

**Table 5:** Mean and standard deviation of oxygen saturation in both groups

**Group** **time**	**Bupivacaine** **(50 patients)**	**Dexmedetomidine+bupivacaine** **(50 patients)**	**Mean difference**	**CI 95%**		**P-value** *
				**Lower**	**Upper**	
Zero	99.2±1.2	97.8±0.8	1.3	0.9	1.7	0.000
5th minute	99.4±0.9	99.5±0.5	-.1	-0.4	0.2	0.527
10th minute	99.5±0.7	99.4±0.7	0.0	-0.2	0.3	0.58
15th minute	99.6±0.5	99.4±0.7	0.2	-0.0	0.5	0.075
30th minute	99.6±0.7	99.2±0.7	0.4	0.1	0.6	0.009
60th minute	99.2±0.9	99±0.8	0.1	-0.1	0.5	0.3
120th minute	98.6±0.9	98.1±0.9	0.4	0.0	0.8	0.014
6th hour	97.9±0.9	97.7±0.8	0.2	-0.1	0.6	0.161
12th hour	97.7±0.9	97.4±0.8	0.2	-0.1	0.5	0.166
18th hour	97.6±0.8	97.5±0.7	0.1	-0.2	0.4	0.524
24th hour	97.7±0.6	97.4±0.6	0.3	0.1	0.5	0.018
P-value	0.000	0.000				

* Independent sample t-test

**Table 6:** Shivering, nausea, and vomiting conditions of patients in both groups

	**Group**	**Bupivacaine** **Number (%)**	**Dexmedetomidine+bupivacaine** **Number (%)**	**P-value** *
Shivering	Without	14(28)	7(14)	0.14
	With	36(72)	43(86)	
Nausea and vomiting	Without	32(64)	31(62)	1
	With	18(36)	19(38)	

* Chi-square

## Discussion

The use of dexmedetomidine as a local anesthetic adjuvant has been increasingly reported to extend the duration of both motor and sensory blockade produced by single Injection neuraxial. (1, 3). The present study was conducted to determine the effect of intrathecal administration of dexmedetomidine plus bupivacaine on analgesia in lower abdominal surgery. The results showed that the mean duration of analgesia was significantly longer in dexmedetomidine plus bupivacaine group than in the bupivacaine group. As a result, adding dexmedetomidine prolongs the duration of analgesia after surgery. 

Based on VAS, the severity of pain in all investigated periods after the onset of pain was significantly lower in the dexmedetomidine plus bupivacaine group than in the bupivacaine group. In a study conducted by Kaya FN et al. in Turkey on 75 ASA I and II patients undergoing spinal anesthesia with bupivacaine, patients were randomly divided into three groups. The first, second, and third groups received intravenous dexmedetomidine, midazolam, and intravenous normal saline before spinal anesthesia, respectively. The results showed that the intravenous administration of dexmedetomidine could increase the duration of bupivacaine block, sedation, and analgesia (10). In a study in India on 60 ASA I- II patients, aged 18-50 years, undergoing lower abdominal surgery, Goupta et al. in 2011 divided the patients into two groups. The first group received intrathecal bupivacaine plus fentanyl, and the second group received intrathecal bupivacaine+dexmedetomidine. On average, patients in the dexmedetomidine group had significantly longer motor and sensory block than those in the fentanyl group (3). In a study conducted by Shukla et al. in India on 90 ASA I and II patients, aged 18-45 years, undergoing lower abdominal and lower limb surgeries, patients were randomly divided into three groups. The first, second, and third groups received bupivacaine plus dexmedetomidine, bupivacaine plus magnesium sulfate, and bupivacaine diluted by saline, respectively. The results showed that the onset and duration of motor and sensory block were longer in the dexmedetomidine group than in the other two groups (2). Al-Mustafa et al. in 2008 conducted a study in Jordan on 66 patients undergoing urologic surgery. In their study, the patients were divided into three groups. The first, second, and third groups received bupivacaine 12.5mg and normal saline, bupivacaine 12.5 mg plus dexamethasone 5μg, and bupivacaine 12.5 mg besides dexamethasone 10 μg, respectively. The results showed that intrathecal dexmedetomidine shortened the onset of motor block and prolonged the regression of motor and sensory block (11). Consistent with similar studies, the present clinical trial showed that the intrathecal administration of dexmedetomidine could prolong the onset of postoperative pain. Moreover, the intrathecal administration of dexmedetomidine reduced the severity of pain in the case group as compared to the control group.

The results showed that despite significant differences between the two groups in examining hemodynamic parameters, such as HR, BP, and OS, in some points of time, they followed a normal decreasing trend. Moreover, the hemodynamic decreasing trend (HR and BP) during the examined period was greater in the dexmedetomidine group than in the dexmedetomidine+bupivacaine group, but not significantly. This difference may be due to the inherent effect of dexmedetomidine (12). Kanazi et al. conducted a study in Lebanon on 60 ASA I and II patients who underwent prostate surgery and had bladder tumor. Patients were divided into three groups using double-blind method. The first, second, and third groups received bupivacaine plus dexmedetomidine, bupivacaine plus clonidine, and bupivacaine alone, respectively. It was observed that dexmedetomidine+clonidine could increase the duration of sensory and motor block of bupivacaine, without causing a drastic change in hemodynamic parameters of the patients (4). AL-Mustafa et al. conducted a study in Jordan on 48 patients undergoing spinal surgery with isobaric bupivacaine. They observed that the intravenous administration of dexmedetomidine could increase the duration of sensory and motor block of intrathecal bupivacaine while hemodynamic stability had been maintained (13). The present study showed that intrathecal dexmedetomidine could not cause significant changes in hemodynamic parameters.

Also, the dexmedetomidine+bupivacaine and bupivacaine groups were not significantly different in terms of shivering; however, it was more severe in the bupivacaine group, so the eight subjects in this group (versus no subject in the dexmedetomidine plus bupivacaine group) needed treatment. Burhanettin Usta et al. in 2011 designed a study in Turkey on 60 ASA I and II patients, aged 18-50 years, undergoing elective minor surgeries under spinal anesthesia. The patients were randomly divided into two groups. The first and the second groups received intravenous dexmedetomidine and intravenous normal saline, respectively. It was observed that dexmedetomidine could significantly reduce shivering in spinal anesthesia without causing any significant complication (14).

The postoperative nausea and vomiting conditions in the two groups were not significantly different; although, fewer patients in the dexmedetomidine group needed metoclopramide treatment. Massad et al. conducted a study on 81 ASA I patients undergoing laparoscopy. Their results showed that adding dexmedetomidine significantly reduced the incidence of postoperative nausea and vomiting (15).

In Conclusion, Administration of dexmedetomedine through the intrathecal route by acting as an adjuvant drug to local anesthetics provided an analgesic effect in postoperative pain without sedation. It could potentiate the effect of the local anesthetic and allow a decrease in the required doses. The present study showed that dexmedetomidine in addition to local anesthetics could prolong the duration of both sensory and motor blockade and postoperative pain management without causing a significant clinical change in patients’ hemodynamic conditions.

**Limitations of this study:**

Trying to gather the exact data in close monitoring during surgery and unreliable patient's information were the major limitations of this study, while this trial like other trials could be influenced by experimenter bias, straw man comparison, unsuccessful randomization and composite outcomes as potential limitations of all clinical trial.
